# Vitamin B12-Impaired Metabolism Produces Apoptosis and Parkinson Phenotype in Rats Expressing the Transcobalamin-Oleosin Chimera in Substantia Nigra

**DOI:** 10.1371/journal.pone.0008268

**Published:** 2009-12-21

**Authors:** Carlos Enrique Orozco-Barrios, Shyue-Fang Battaglia-Hsu, Martha Ligia Arango-Rodriguez, Jose Ayala-Davila, Celine Chery, Jean-Marc Alberto, Henry Schroeder, Jean-Luc Daval, Daniel Martinez-Fong, Jean-Louis Gueant

**Affiliations:** 1 Department of Physiology, Biophysics and Neuroscience, Centro de Investigación y Estudios Avanzados del Instituto Politécnico Nacional (CINVESTAV), Mexico City, Mexico; 2 Inserm U954, Faculté de Médecine, Nancy-Université, Nancy, France; 3 Institut national de la recherche agronomique (INRA), URAFPA, Nancy-Université, Nancy, France; AgroParisTech, France

## Abstract

**Background:**

Vitamin B12 is indispensable for proper brain functioning and cytosolic synthesis of S-adenosylmethionine. Whether its deficiency produces effects on viability and apoptosis of neurons remains unknown. There is a particular interest in investigating these effects in Parkinson disease where Levodopa treatment is known to increase the consumption of S-adenosylmethionine. To cause deprivation of vitamin B12, we have recently developed a cell model that produces decreased synthesis of S-adenosylmethionine by anchoring transcobalamin (TCII) to the reticulum through its fusion with Oleosin (OLEO).

**Methodology:**

Gene constructs including transcobalamin-oleosin (TCII-OLEO) and control constructs, green fluorescent protein-transcobalamin-oleosin (GFP-TCII-OLEO), oleosin-transcobalamin (OLEO-TCII), TCII and OLEO were used for expression in N1E-115 cells (mouse neuroblastoma) and in *substantia nigra* of adult rats, using a targeted transfection with a Neurotensin polyplex system. We studied the viability and the apoptosis in the transfected cells and targeted tissue. The turning behavior was evaluated in the rats transfected with the different plasmids.

**Principal Findings:**

The transfection of N1E-115 cells by the TCII-OLEO-expressing plasmid significantly affected cell viability and increased immunoreactivity of cleaved Caspase-3. No change in propidium iodide uptake (used as a necrosis marker) was observed. The transfected rats lost neurons immunoreactive to tyrosine hydroxylase. The expression of TCII-OLEO was observed in cells immunoreactive to tyrosine hydroxylase of the substantia nigra, with a superimposed expression of cleaved Caspase-3. These cellular and tissular effects were not observed with the control plasmids. Rats transfected with TCII-OLEO expressing plasmid presented with a significantly higher number of turns, compared with those transfected with the other plasmids.

**Conclusions/Significance:**

In conclusion, the TCII-OLEO transfection was responsible for apoptosis in N1E-115 cells and rat *substantia nigra* and for Parkinson-like phenotype. This suggests evaluating whether vitamin B12 deficit could aggravate the PD in patients under Levodopa therapy by impairing S-adenosylmethionine synthesis in *substantia nigra*.

## Introduction

Vitamin B12 (cobalamin) deficiency in human has long been linked to pernicious anemia. However, this is not the only ailment caused by the lack of cobalamin. A neurological counterpart of this anemia is the SCD, subacute combined degeneration [1]. Vitamin B12 deficiency leads to memory disturbance, cognitive decline and dementia [2]. With few exceptions, the pathological consequences of vitamin B12 deficiency have been based on the only two known vitamin B12 dependent biochemical reactions in the mammalian cells, involving the mitochondrial L-methylmalonyl-coenzyme A mutase (MMCM; EC 5.4.99.2) and the cytoplasm homocysteine (Hcy) methyltransferase, also referred as methionine synthase (MS; EC 2.1.1.13) [2]. Inferences made are thus based on the two direct consequences of lacking B12: the accumulation of methylmalonic acid and Hcy. Hcy is a metabolite of the essential amino acid methionine that can either be re-methylated to methionine by methionine synthase, an enzyme that requires folate (vitamin B9) and vitamin B12, or be catabolized by cystathionine beta-synthase (CBS) to generate cysteine [3]. Methionine is converted into S-adenosylmethionine (SAM), which is the universal methyl donor in the trans-methylation reactions involved in epigenetic functions, cell metabolism, and neurotransmitter synthesis and catabolism. Despite the wealth of information available for the pathology of cobalamin deficiency, it remains difficult to explain the molecular mechanisms that can bring about all the neurological manifestations observed, since their identification is limited by the lack of specific experimental cell and animal models [2]. In particular, whether the vitamin B12 deficiency produces effects on viability and apoptosis of dopaminergic neurons remains unknown. There is a particular interest in investigating these effects in Parkinson disease where Levodopa treatment is known to increase the consumption of S-adenosylmethionine [4].

Several models of vitamin B12 deprivation have been developed *in vitro* and *in vivo* to determine the biochemical and molecular mechanisms of the cell impairment caused by vitamin B12 deficiency. The *in vitro* models consist of the use of culture media lacking vitamin B12 or supplemented with Hcy [5]–[7]. The models in experimental animals follow various designs and strategies. One chronically provides diets without vitamin B12 and methyl donors to pregnant rats and evaluates afterwards alterations in the pups of the treated dams [8], [9]. The other is to provide diets lacking vitamins or supplemented with Hcy to adult animals [10], [11]. The neurological effects of the deficient diet has been documented in one of these in vivo models [8]. The deficiency of dams in methyl precursors, folate, vitamin B12 and choline contributes to an impaired cognition, and at the tissue level to the apoptosis linked with Hcy accumulation and atrophy of the CA1 hippocampus atrophy in pups [8], [12]. The deficiency in vitamin B12, folate and vitamin B6 also produces a rarefaction of hippocampus microvasculature in adult mice [13]. Gastrectomy in rats has been used to abolish the secretion of intrinsic factor, the protein responsible for the intestinal absorption of vitamin B12. Beside the B12 deficiency, this model produces dramatic effects related with a central and peripheral neuropathy, concurrently with a denutrition and a deficit in other important nutrients such as iron, folic acid, and vitamin E [2], [14], [15].

To cause deprivation of vitamin B12, we have recently developed a cell model deficient in B12 by anchoring transcobalamin (TCII) to the endoplasmic reticulum through its fusion with Oleosin (OLEO), a plant protein localized to lipid droplets and endoplasmic reticulum of plant cells [16]–[18]. TCII is the plasmatic transporter of vitamin B12 with high affinity and specificity for B12 binding [18]. OLEO is a plant protein anchoring onto the surface of seed oil bodies by its central hydrophobic domain, which was used to target the secreted TCII protein to the intracellular membranes of reticulum, in mammalian cells [16]–[18]. These plasmids were transfected into various cell lines, including N1E-115 neuroblastoma cells. TCII-OLEO was a very efficient chelator of cobalamin while OLEO-TCII transfected cells were no different from the either wild type non-transfected cells or cells expressing TCII alone. This suggested that integration of TCII in the C-terminal of the OLEO impaired the B12 binding capacity of the chimer protein [16], [19].

The use of anti-TCII antibodies produces vitamin B12 deficiency and impairs the growth of leukemia cells by a mechanism related with cell viability [20]. Whether B12 selective deficiency produces effects on viability and apoptosis of brain tissue remain unknown, probably because of experimental limitations. Indeed, studies in CNS-related B12 deficit should consider not only the proliferated state of neurons, but also the fully differentiated mature CNS, given the dramatic neurological effects of B12 deficiency in the elderly [2]. Cell models such as N1E-115 are tyrosine hydroxylase (TH) expressing cells adapted for evaluating the effects of B12 in proliferating cells but not those in mature CNS since once these cells reach fully differentiation state, they rapidly become detached from the culture dishes, reflecting the cell death. To encompass this problem, we therefore aimed to use our TCII-OLEO chimera model for performing targeted in vivo transfection of the plasmid constructs in the *substantia nigra* of adult rats and compared the viability effects with those in N1E-115 cells. We studied the functional consequences of the TCII-OLEO transfection in the targeting tissue by comparing the behavior of the rats after unilateral transfection with the different plasmid constructs.

## Results

### Transgenic Expression

**Figure 1:**
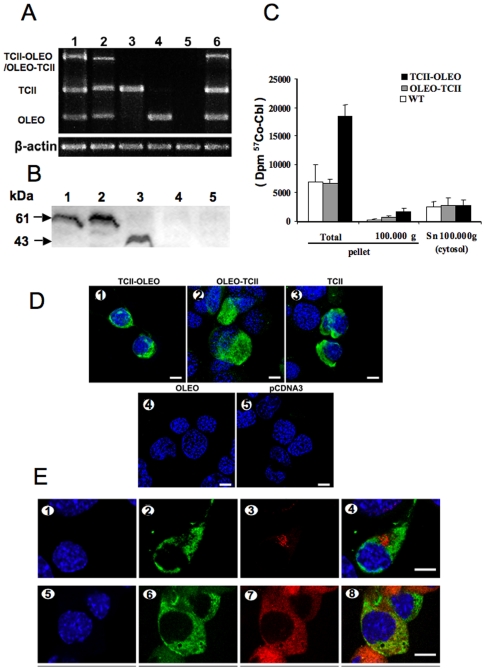
Expression of transcobalamin/oleosin chimeric proteins in transfected N1E-115 cells. A: Transgenic expression in N1E-115 cells at 48 h after transfection using lipofectamine. The cells were transfected with one of the following plasmids: pCMV-TCII-OLEO coding for transcobalamin-oleosin (lane 1), pCMV-OLEO-TCII coding for oleosin-transcobalamin (lane 2), pCMV-TCII coding for transcobalamin II (lane 3), pCMV-OLEO coding for oleosin (lane 4), pCDNA3 (lane 5), and pCMV-GFP-TCII-OLEO coding for GFP-TCII-OLEO (lane 6). The housekeeping gene was β-actin. The size in bp of the amplified products was 1347 for transcobalamin-oleosin (TCII-OLEO), 1240 for oleosin-transcobalamin (OLEO-TCII), 551 for transcobalamin II (TCII), 275 for oleosin (OLEO), and 349 for β-actin. B: Western blotting of homogenate of N1E-115 cells transfected with the various plasmids. From lane 1 to 6: homogenates from cells transfected with pCMV-TCII-OLEO, pCMV-OLEO-TCII, pCMV-TCII, pCMV-OLEO, empty plasmid, pCMV-GFP-TCII-OLEO, respectively. C: Vitamin B12 binding capacity in transfected cells. ^57^Co-labeled Cobalamin (Cbl, ∼300 µCi per µg) was incorporated into culture medium (30,000 dpm/mL) for three days. The total amount of radioactivity taken by each cell lines was measured in pellets and supernatants. Mean and S.E.M. are indicated. D: Indirect immunofluorescence of TCII in N1E-115 cells transiently transfected with lipofectamine. The four constructs and the empty plasmid were tranfected in N1E-115 cells (1–5). The immunofluorescence was done with a goat polyclonal antibody to TCII and a donkey antigoat IgG fluorescein labeled. Cell nuclei were counterstained with Hoechst 33258. Calibration bars = 10 µm. E: Confocal analysis showing co-localization of the protein GFP-TCII-OLEO with endoplasmic reticulum in transfected N1E-115 cells. The cells were transfected with the plasmid pCMV-GFP-TCII-OLEO coding for GFP-transcobalamin-oleosin (GFP-TCII-OLEO), using lipofectamine. Cell nuclei were counterstained with Hoechst 33258 (1, 5). Co-localization was evidenced with fluorescence from GFP (2, 6), immuno-fluorescence with a mouse monoclonal antibody to the human golgin-97 (3) or a rabbit polyclonal antibody to calreticulin (7) and merge fluorescence (4,8). The secondary antibodies include a donkey IgG anti-mouse TRITC labeled or a donkey IgG anti-rabbit TRITC labeled. Calibration bars = 20 µm.

The transgenic expression in N1E-115 cells was assessed by RT-PCR, western blot, and immunofluorescence assays. RT-PCR showed the expected amplified product of 1347 bp for TCII-OLEO, 1240 bp for OLEO-TCII, 551 bp for TCII, and 275 bp for OLEO (Fig. 1A). The recombinant chimera and TCII had the expected size in western blotting with anti-TCII, while no TCII protein was detected after the transfection with either the plasmid pCMV-OLEO or the empty plasmid (Fig. 1B). The TCII-OLEO transfected cells had a dramatically higher binding with labeled B12 in the membrane fraction, compared with other transfected cells, when ^57^Co-labeled Cobalamin was incorporated into culture medium for three days (Fig. 1C). The expression of the recombinant proteins was also evidenced by the indirect immunofluorescence in the transfected N1E-115 cells, using an anti-TCII polyclonal antibody (Fig. 1D).

### Intracellular Location of the Protein Fusion TCII-OLEO

To show the functionality of OLEO as an anchoring protein to immobilize TCII in cell membrane structures after their transgenic expression, we made co-location assays of the expression of the triple fusion protein GFP-TCII-OLEO by immunostaining of either calreticulin, a protein of ER membrane, or golgin 97, a protein of Golgi apparatus, as described previously in COS-7 cells [16]. Confocal microscopy analysis showed that the fluorescence of the triple fusion protein GFP-TCII-OLEO co-located with calreticulin rather than with golgin 97 (Fig. 1E), suggesting the anchoring of the chimeric protein mainly on ER membrane.

### Cell Viability

**Figure 2:**
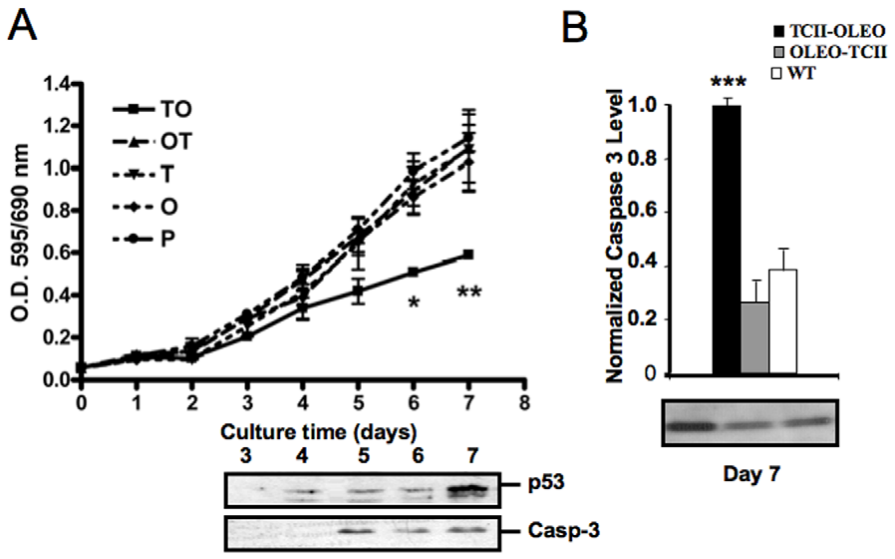
Viability and apoptosis of N1E-115 cells stably transfected with different plasmids. The plasmids were pCMV-TCII-OLEO coding for transcobalamin-oleosin (TCII-OLEO), pCMV-OLEO-TCII coding for oleosin-transcobalamin (OLEO-TCII), pCMV-TCII coding for transcobalamin II (TCII), pCMV-OLEO coding for oleosin (OLEO), and pCDNA3. A: Cell viability and apoptosis among growth delay in proliferated state was monitored by the absorbance of formazan dye resulting from enzymatic metabolism of MTT by the mitochondrial dehydrogenase. The no reagent blank was MTT alone (0.5 mg/mL in culture medium). Western blot analysis of the temporal course of p53 in cells transfected with pCMV-TCII-OLEO was made by using a mouse anti-p53 antibody and a mouse anti-actin antibody, which were revealed with a goat anti-mouse IgG coupled to peroxidase. Cleaved Caspase-3 was identified by a rabbit anti-caspase-3 polyclonal antibody and a donkey anti-rabbit secondary antibody (labeled with peroxidase). B: Western blotting of cleaved Caspase-3 at Day 7 of proliferate state. The mean±S.E.M. were obtained from three independent experiments made in triplicate. Two-way ANOVA and Bonferoni post-test analyzed statistical differences from the control groups. *, *P*<0.05; **, *P*<0.01; ***, *P*<0.001.

We explore whether the expression of the anchoring chimeric proteins in membrane structures would lead to cytotoxic effects possibly associated with vitamin B12 deprivation. Viability assays with MTT (3-(4,5-dimethylthiazol-2-yl)-2,5-diphenyl tetrazolium bromide) were made in murine neuroblastoma N1E-115 cells stability transfected with each of the plasmids of interests. Only the transfection of TCII-OLEO significantly affected the cell viability when compared with the transfection of OLEO or TCII plasmids (Fig. 2A). The statistical significance was between the sixth and seventh days of culture. Interestingly, cells transfected with OLEO-TCII showed kinetics similar to that of the control cells, suggesting that the expression of this fusion protein was not cytotoxic.

### Apoptosis and Necrosis in Stably Transfected Cells

**Figure 3:**
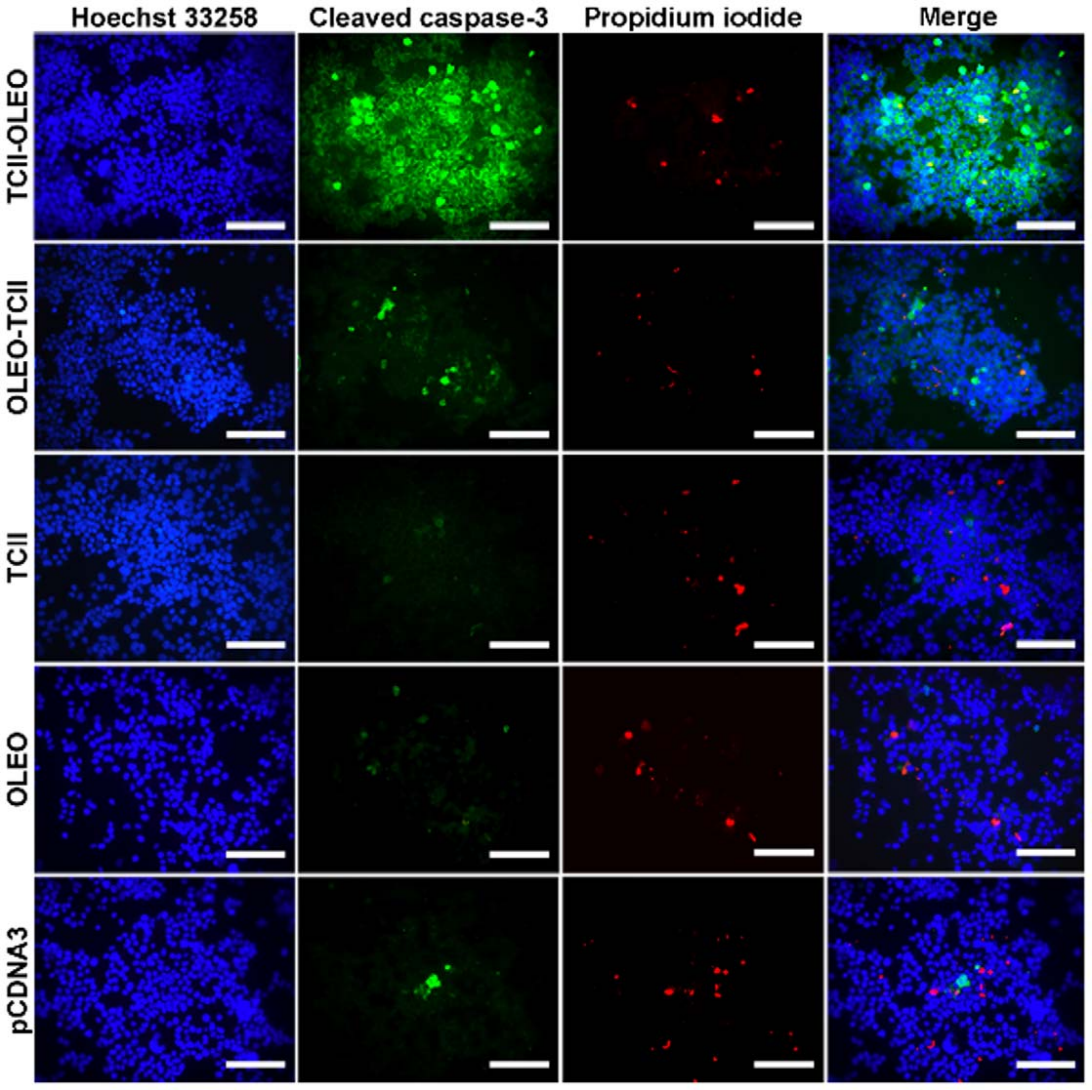
Analysis of apoptosis in N1E-115 cells stably transfected with different plasmids. The plasmids were pCMV-TCII-OLEO coding for transcobalamin-oleosin (TCII-OLEO), pCMV-OLEO-TCII coding for oleosin-transcobalamin (OLEO-TCII), pCMV-TCII coding for transcobalamin II (TCII), pCMV-OLEO coding for oleosin (OLEO), and pCDNA3. The immunofluorescence was done with a rabbit polyclonal antibody to cleaved Caspase-3 and a donkey antirabbit IgG fluorescein labeled. Before fixation, cells were incubated with 4 µM propidium iodide for 10 min. Cell nuclei were counterstained with Hoechst 33258. Calibration bars = 100 µm.

To determine the type of cell-death in the stably transfected cells, we used western blotting and immunofluorescence of cleaved Caspase-3, the active form of this cysteine protease, and western blotting against p53 as apoptosis markers (Fig. 2 and 3). The uptake of propidium iodide uptake was used as a necrosis marker. A significant increase in the immunoreactivity to both p53 and cleaved Caspase-3 was observed after 5 days of culture in N1E-115 cells transfected with TCII-OLEO plasmid (Fig. 2 and 3), while no change was observed in earlier growth delay (Fig. 2). The immunoreactivity of cleaved Caspase-3 was much higher in TCII-OLEO expressing cells than in cells transfected with the other constructs (Fig. 2B and 3). No difference was observed in the uptake of propidium iodide when compared with the control values (Fig. 3), indicating that apoptosis was the main type of cell death caused by the expression of TCII-OLEO protein. Consistently with the viability assays, the transfection of OLEO-TCII plasmid did not increase either cleaved Caspase-3 or propidium iodide uptake (Fig. 2B and 3).

### Transfection of the Plasmids in Rats

**Figure 4:**
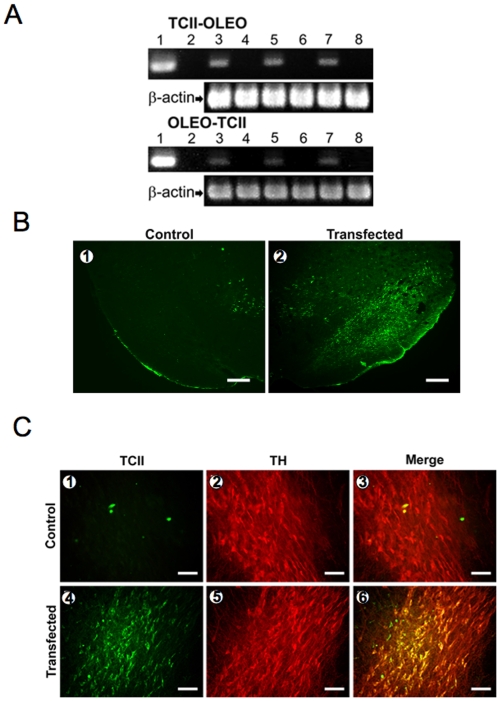
Expression of transcobalamin II/oleosin (TCII/OLEO) chimeric proteins in rats 60 days after transfection with the NTS-polyplex. A: RT-PCR from the plasmid transcripts in the *substantia nigra* of rats. A group of rats (n = 3) was transfected with the plasmid pCMV-TCII-OLEO and another (n = 3) with the plasmid pCMV-OLEO-TCII. RT-PCR amplified a fragment of 380 bp for TCII-OLEO, a fragment of 394 for OLEO-TCII, and a fragment of 349 for β-actin, the internal control. Lane 1 corresponds to the amplified fragment from the plasmid (positive control). Lane 2 is a PCR in the absence of plasmid or cDNA (negative control). The amplified product from the transfected substantia nigra of each rat corresponds to the lanes 3, 5, and 7, and the lanes 4, 6, and 8 show the RT-PCR outcome from the non-transfected side. B: GFP immunofluorescence in the rat *substantia nigra* transfected with pCMV-GFP-TCII-OLEO. The pCMV-GFP-TCII-OLEO encodes for the fusion protein green fluorescent protein-transcobalamin-oleosin (GFP-TCII-OLEO). The immunofluorescence was done with a mouse monoclonal antibody to GFP and a donkey antimouse IgG fluorescein labeled. Representative micrographs of coronal section of control substantia nigra (1) and transfected substantia nigra (2) of the same rat are presented. Calibration bars = 100 µm. C: Double immunofluorescence against TCII and tyrosine hydroxylase (TH) in the *substantia nigra* of rats. The neurons were transfected with NTS-polyplex with pCMV-TCII-OLEO coding for transcobalamin-oleosin (TCII-OLEO). Slices from mesencephalon (40 µm) were immunostained at 7-day after transfection. The primary antibodies were a goat polyclonal anti-TCII and a mouse monoclonal anti-TH. The secondary antibodies were a donkey antigoat IgG fluorescein labeled and a donkey antimouse IgG rhodamine labeled. Representative micrographs of coronal section of control *substantia nigra* (1–3) and transfected *substantia nigra* (4–6) of the same rat are presented. Calibration bars = 50 µm.

**Figure 5:**
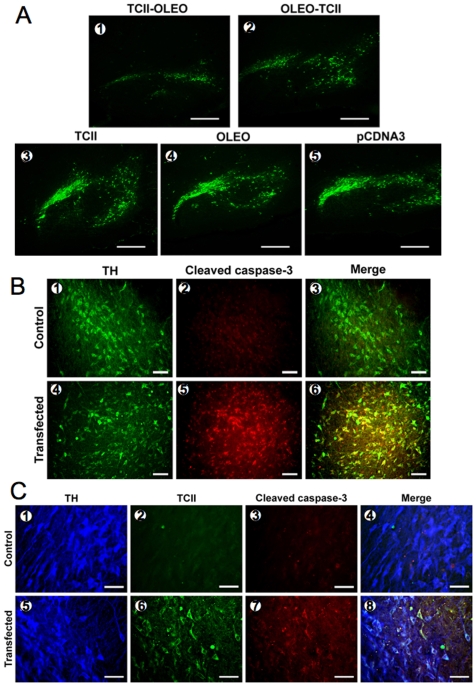
Apoptosis of tyrosine hydroxylase (TH) immunoreactive cells in the *substantia nigra* of rats transfected with several plasmids. A: TH-immunoreactive neurons after transfection. The neurons were transfected with NTS-polyplex with one of the following plasmids, pCMV-TCII-OLEO coding for transcobalamin-oleosin (TCII-OLEO, 1), pCMV-OLEO-TCII coding for oleosin-transcobalamin (OLEO-TCII, 2), pCMV-TCII coding for transcobalamin II (TCII, 3), pCMV-OLEO coding for oleosin (OLEO, 4), and the pCDNA3, the empty plasmid (5). Mesencephalon slices (40 µm) were immunostained at 2-month after transfection with a mouse monoclonal antibody to TH and a donkey antimouse IgG fluorescein labeled. Representative micrographs of sagital section of the rat mesencephalon are presented. Calibration bars = 200 µm. B: Apoptosis in TH-immunoreactive neurons after transfection with the plasmid pCMV-TCII-OLEO. Representative micrographs of the *substantia nigra* (with double immunostaining at 15-day after transfection) are presented. The primary antibodies were a mouse monoclonal antibody to TH, and a rabbit polyclonal antibody to cleaved Caspase-3. The secondary antibodies included a donkey anti-mouse IgG FITC labeled (1 and 4), and a donkey anti-rabbit IgG rhodamine labeled (2 and 5). Representative micrographs of coronal section of control *substantia nigra* (1–3) and transfected *substantia nigra* (4–6) of the same rat are presented. Scale bars = 50 µm. C: Apoptosis in TH immunoreactive neurons expressing the TCII-OLEO chimera. Representative micrographs of the *substantia nigra* with triple immunostaining at 15-day after transfection. The primary antibodies were a mouse monoclonal antibody to TH, a goat polyclonal antibody to TCII, and a rabbit polyclonal antibody to cleaved Caspase-3. The secondary antibodies were a donkey anti-mouse IgG AMCA labeled (1 and 5), a donkey antigoat IgG fluorescein labeled (2 and 6), a donkey anti-rabbit IgG rhodamine labeled (3 and 7). Representative micrographs of coronal section of control *substantia nigra* (1–4) and transfected *substantia nigra* (5–8) of the same rat are presented. Scale bars = 50 µm.

pCMV-GFP-TCII-OLEO, pCMV-TCII-OLEO, pCMV-OLEO-TCII, pCMV-OLEO, pCMV-TCII, and pCDNA3 were transfected *in vivo* into nigral neurons of adult rats, using the neurotensin (NTS)-polyplex targeting system. The expression of TCII-OLEO and OLEO-TCII were shown in homogenates of the *substantia nigra* 60 days after the transfection using RT-PCR (Fig. 4A). The expression of GFP-TCII-OLEO in *substantia nigra* was observed 7 days after the transfection (Fig. 4B). The immunodetection of TCII was observed in TH-immunoreactive neurons of rats transfected with pCMV-TCII-OLEO (Fig. 4C). The TCII-OLEO-transfected rats lost TH-immunoreactive neurons (Fig. 5A). A weak effect was observed in OLEO-TCII transfected animals, while the activity of rats transfected with either the TCII or OLEO plasmids was similar to that of the animals transfected with the empty plasmid pCDNA3 (Fig. 5A). Co-location of the immunoreactivities of TH and cleaved Caspase-3 was evidenced in the *substantia nigra* of the TCII-OLEO-transfected animals (Fig. 5B). In addition, the TH-immunoreactive neurons expressing the cleaved Caspase-3 were also those expressing the TCII-OLEO chimera (Fig. 5C).

### Behavior and Methamphetamine-Induced Turning Test in Transfected Rats

**Figure 6:**
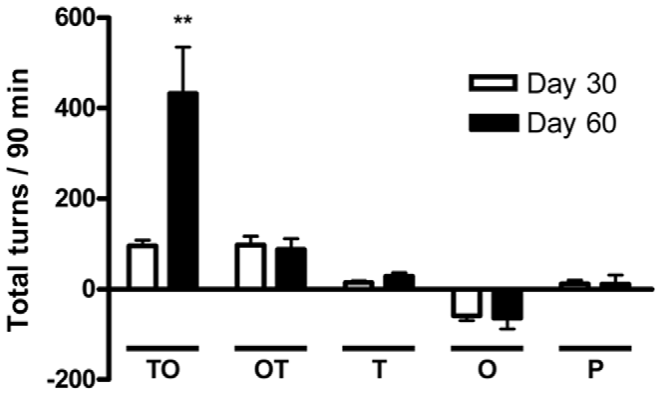
Methamphetamine-induced turning behavior in rats transfected with several plasmids. The plasmids were pCMV-TCII-OLEO coding for transcobalamin-oleosin (TO), pCMV-OLEO-TCII coding for oleosin-transcobalamin (OT), pCMV-TCII coding for transcobalamin II (T), pCMV-OLEO coding for oleosin (O), and pCDNA3 (P). The values are the mean±SEM of 3 animals per group. ** = Significantly different from control groups. *P*<0.01, repeated-measures two-way ANOVA and Bonferroni post-test.

The rats transfected with pCMV-TCII-OLEO presented with a significantly higher number of ipsilateral turns, compared with the animals transfected with either the pCMV-OLEO-TCII, pCMV-TCII pCMV-OLEO or the empty pCDNA3 plasmids (Fig. 6). The number of ipsilateral turns reported in OLEO-TCII rats was not statistically significant from that reported in the other control groups. No difference among the different groups of transfected rats was reported in the open field test (data not shown).

## Discussion

The transgenic expression of TCII-OLEO, OLEO-TCII, TCII, and OLEO in N1E-115 cells was ascertained by three complementary experiments, RT-PCR of mRNAs, western blot and confocal immunofluorescence analysis of the protein products. In addition, the fusion protein GFP-TCII-OLEO was anchored mainly in the membrane of endoplasmic reticulum of the transfected N1E-115 cells, as previously showed for COS-7 cells [16]. This was supported by the co-location of the fusion protein GFP-TCII-OLEO with the immunostaining of calreticulin, a calcium pump in ER membrane, and the absence of co-location with the immunostaining of golgin-97, a marker Golgi apparatus [21], [22]. We also confirmed that the TCII-OLEO chimera produced a significant binding of vitamin B12 in the cellular membrane fraction of the transfected cells. In contrast, the OLEO-TCII chimera produced the same vitamin B12 binding as the TCII- and OLEO-transfected cells, as previously showed [16], [23]. N1E-115 cells expressing TC-OLEO, but not those expressing OLEO-TC, have an impaired cellular metabolism of B12, with decreased SAM, and increased Hcy and methylmalonic acid. Compared with OLEO-TC expressing cells, the TC-OLEO expressing cells had a decreased proliferation rate, an increased expression of p38 and a decreased expression of ERK 1/2 [23]. This may explain that, of the five plasmids individually assessed, only the transfection of pCMV-TCII-OLEO caused apoptotic cell-death. The increased immunoreactivity in cleaved Caspase-3, the active form of this cysteine protease, and the absence of propidium iodide uptake supported the presence of apoptosis and absence of necrosis in the TCII-OLEO expressing cells. No differences in cell viability and apoptosis were observed between the cells expressing OLEO-TCII, TCII or OLEO, or transfected with the empty plasmid, showing that the apoptotic effect was produced neither by OLEO nor TCII. We showed recently that the stable transfection of N1E-115 cells with the TCII-OLEO plasmid leads to decreased conversion of cyano-cobalamin to methyl-cobalamin, the co-factor of methionine synthase, and this is accompanied by a subsequent decrease in the activity of methionine synthase and an increase of Hcy and reduced SAM [16], [23]. These effects on intracellular metabolism are not observed in the cells transfected with the other plasmids.

The *in vivo* transfection of rats with the TCII-OLEO expressing plasmid produced similar effects as those observed with N1E-115 transfected cells, with the loss of neurons expressing TH and an increased expression for cleaved Caspase-3 expression in the *substantia nigra*. Taken together, our data strongly suggest that the intracellular sequestration of vitamin B12 by the TCII-OLEO protein anchored to ER might be the cause of the apoptotic cell-death, by a mechanism related with vitamin B12 impaired metabolism.

The nutritional in vivo models of global B12 deficiency are not adapted for investigating the Parkinson-like phenotype. In these models, the deficient diet would theorically produce bilateral effects on substantia nigra, in addition to other effects on other brain regions and to peripheral neuropathy, anemia and muscular weakness [8]–[15]. These effects may complicate the interpretation of behavior abnormalities registered in the deficient animals. In contrast, our experimental approach allowed the evaluation of the behavior consequences produced by the selective and unilateral lesions of *substantia nigra*, where methamphetamine is expected to stimulate only the intact non-transfected cells. Methamphetamine-induced turning in rats transfected with several plasmids provided a nice confirmation of the consequence of the loss of dopaminergic neurons in the *substantia nigra* of the TCII-OLEO transfected rats. The number of ipsilateral turns was 4.5-fold higher in TCII-OLEO transfected animals, compared with those expressing the OLEO-TCII chimera. The difference between the TCII-OLEO transfected rats and the other groups of transfected rats was only evidenced after 60 days. This could be related with the time needed in vivo for producing a B12 deficiency in transfected tissue, as a function of the existing store of B12 in brain [8]. The turns were ipsilateral to the side of the transfection, in consistence with the amphetamine stimulation of the intact side of the *substantia nigra* [24].

Various effects of vitamin B12 deficiency may explain the apoptotic effect observed *in vitro* and *in vivo* in lived transfected rats. Apoptosis could be related with the TNF pathway as previous observations made in humans deficient in B12 showed an increased level TNF in the spinal fluid [2], [25]. The decreased activity of methionine synthase leads to an impaired re-methylation and an accumulation of Hcy [16]. Accumulation of Hcy and deficit in methyl groups produce apoptotic cell death, as evidenced in several animal models and in vitro [6]–[9].

Plasma Hcy, vitamin B12 and folate and the C677T mutation in MTHFR are associated with Parkinson disease (PD) [26]–[29]. Hyperhomocysteinemia has been linked to PD by oxidative effect [30]–[33] and influences dopamine synthesis [34], while Levodopa treatment promotes hyperhomocysteinemia [35]–[37]. The hyperhomocysteinemia in Levodopa treated PD patients could be related with the increased consumption of SAM in the O-methylation of Levodopa, catalyzed by catechol-O-methyltransferase (COMT) [38]. Consequently, folate and vitamin B12 supplementations reduce the hyperhomocysteinemia in PD patients on Levodopa therapy [35]. Our data suggest that the deficiency in vitamin B12 could have dramatic effect on the dopaminergic cells, in patients under Levodopa therapy, considering that (i) B12 deficiency decreases the cellular content in SAM, as has been showed in B12 deficient N1E-115 cells (ii) Levodopa enhances the cellular consumption of SAM, a metabolic condition that may accentuate the apoptotic effect of B12. Given the relative frequency of vitamin B12 deficiency in the elderly [39], [40], our data suggest a further evaluation of the cellular, tissular and functional consequences of Levodopa in case of B12 deficiency, using the TCII-OLEO cells and our model of transfected rat with Parkinson-like phenotype.

In conclusion, the cellular impaired metabolism of vitamin produced by TCII-OLEO transfection is responsible for apoptosis in N1E-115 cells and in rats and for Parkinson-like phenotype in the transfected rats. This suggests evaluating the consequences of vitamin B12 deficit in PD patients under Levodopa therapy.

## Methods

### Plasmids

Three plasmids coding for the fusion protein between the human TCII and the plant OLEO were obtained by cloning their coding sequences in sense orientation into the BamHI–XbaI site of the mammalian expression vector pCDNA3, under the transcriptional control of CMV promoter as recently described [16]. The plasmid pCMV-TCII-OLEO (7.2 kb) codes for the protein TCII-OLEO whereas pCMV-OLEO-TCII (7.1 kb) codes for the protein OLEO-TCII. The plasmid pCMV-GFP-TCII-OLEO (7.9 kb) encodes for the fusion protein green fluorescent protein (GFP)-TCII-OLEO, which was used as reporter protein. The control plasmids were pCMV-TCII (6.6 kb) coding for TCII, the pCMV-OLEO (5.9 kb) coding for OLEO, and the empty plasmid pCDNA3 (5.4 kb).

### Transfection *In Vitro*

Neuroblastoma N1E-115 cells, used in stable expression assays, were obtained from the American Type Culture Collection, and cultured in DMEM supplemented with 10% fetal bovine serum (FBS) and penicillin-streptomycin (100 µg/mL of each). Cell cultures were kept at 37°C under a 5% CO_2_ atmosphere. Transient and stable transfections were preformed as described recently [16]. N1E-115 cells seeded in 6-well dishes were selected with 800 µg/mL of G418 (Sigma-Aldrich, St. Louis, MO, US) for 15 days, and then transferred to 24-well dishes for viability studies. Stably selected N1E-115 cells seeded on 12-mm microscope cover glasses were used for cleaved Caspase-3 immunostaining and propidium iodide incorporation.

### Synthesis of NTS-Vector and NTS-Polyplex Formation

Neurotensin (NTS)-polyplex is a non-viral system for the targeted gene delivery to cells that express and internalize the high-affinity NTS receptor (NTSR1) [41]–[44]. NTS-polyplex results from the electrostatic binding of NTS-FP-poly-L-Lysine (PLL) conjugate (the NTS-vector) with a plasmid DNA (pDNA) previously bound with PK [45]. The detailed procedure of NTS-vector synthesis was reported previously [42], [43]. Briefly, NTS (ELYENKPRRPYIL; Sigma, St. Louis, MO, USA) and FP (GLFEAIAEFIEGGWEGLIEGCAKKK; purity >90%; SynPep, Dublin, CA, USA) were cross-linked with PLL (48 kDa mean molecular mass; Sigma, St. Louis, MO, USA) using LC-SPDP as the cross-linker [42]. Suitable gel-filtration chromatography was used to purify the SPDP derivatives and the NTS–FP–SPDP–PLL conjugate (the NTS-vector). This conjugate was concentrated to 1 mL, further dialyzed against phosphate buffered saline solution (PBS; 8.1 mM Na_2_HPO_4_, 1.2 mM KH_2_PO_4_, 138 mM NaCl, 2.7 mM KCl, pH 7.4), and sterilized by filtration.

The NTS-polyplexes were formed by electrostatically binding the NTS-vector and the mutant Vp1 SV40 KP (MAPTKRKGSCPGAAPNKPK; 90% purity; Synpep Corp., Dublin, CA, USA) to different pDNAs at optimum molar ratio [44]. First, the KP was electrostatically bound to pDNA to form the KP-pDNA complex. The KP and the pDNA were dissolved in serum-free DMEM. Similar amounts of 6 nM pDNA were incubated with increasing amounts of KP (3, 6, 9, 12, 15, 18, 21, 24 µM) for 30 min at room temperature and then subjected to 0.8% agarose gel electrophoresis as described previously [44], [45]. Since the KP concentration of 9 µM for pCMV-TCII-OLEO and 6 µM for pCMV-OLEO-TCII, pCMV-OLEO, pCMV-TCII and pCDNA3 do not saturate the anionic charges of pDNA (6 nM), these concentrations were selected to form the NTS-polyplex.

NTS-polyplexes were formed with a constant concentration of KP-pDNA complexes and increasing concentrations of the NTS-vector (18, 36, 54, 72, 90, 108, 126, 144, 162, 180, 198, 216, 234 nM). The reaction mixtures were incubated for 30 min at room temperature and then subjected to 0.8% agarose gel electrophoresis as described previously [44], [45]. The concentrations of NTS-vector producing the compete retention of KP-pDNA complex in the gel well was considered as the optimum molar ratio [44], [45]. Those concentrations were 180 nM for pCMV-TCII-OLEO and pCMV-OLEO-TCII, 162 nM for pCMV-TCII and pCMV-OLEO, and 126 nM for pCDNA3. The NTS-polyplex were injected 5X concentrated. The final concentration of each component was: plasmid DNA, 30 nM for all the constructs; KP, 45 µM for pCMV-TCII-OLEO and 30 µM for pCMV-OLEO-TCII, pCMV-OLEO, pCMV-TCII and pCDNA; NTS-vector, 900 nM for pCMV-TCII-OLEO and pCMV-OLEO-TCII, 810 nM for pCMV-TCII and pCMV-OLEO, and 630 nM for pCDNA.

### Transfections In Vivo

Adult male Wistar rats (weighing 210–230 gr at the onset of experiment), bred in our facilities, were maintained under constant room temperature (23°C), and light–dark cycle (12-12 h), with food and water ad libitum. All procedures were in accordance with the Mexican legislation (NOM-062-ZOO-1999; SAGARPA), based on the Guide for the Care and Use of Laboratory Animals, NRC. The CINVESTAV Committee for animal care and use (IACUC) approved and supervised our experimental procedures (authorization #0109-02). All efforts were made to minimize animal suffering. Each rat was anesthetized by an intraperitoneal injection of chloral hydrate (350 mg/kg) and placed in a stereotaxic instrument (Model 51600, Stoelting; Wood Dale, ILL, USA) with the incisor bar 5.5 mm below the interaural line. After cranial trepanation, 3 µL of the different NTS-polyplexes were unilaterally microinjected into the dorsal border of left substantia nigra at the coordinates AP, −4.6 mm from bregma; ML, +1.5 mm from the interparietal suture; DV, −6.8 mm from dura mater. The solution was injected at a flow rate of 0.1 µl/min using a microsyringe (Hamilton Company, Reno, Nevada, USA) and an automatic microperfusion pump (Stoelting, Wood Dale, IL, USA). After surgery, all animals were injected with monohydrate of Cefalexin (10 mg/kg, im) to prevent infection [41], [43], [44].

### Reverse Transcription-Polymerase Chain Reaction

Reverse transcription-polymerase chain reaction (RT-PCR) was used to show mRNA expression in both stably transfected N1E-115 cells and rat substantia nigra. RNAs was extracted with Trizol (Invitrogen Corporation Carlsbad, CA, USA) from 7×10^5^ N1E-115 cells or form a complete substantia nigra, quantified by spectrophotometry at 260 nm, and analyzed by 2%-agarose gel electrophoresis. The RNA preparations were treated with RNase-free DNase before their use in the reverse transcription. Five µg of total RNA was transcribed with SuperScript II reverse transcriptase (200 U) using 0.1 µg of oligo dT (Invitrogen Corporation, Carlsbad, CA, USA). One µL of the reverse transcribed product were amplified in a temperature gene cycler (Gene Amp PCR System 9700; Applied Biosystems, Foster city, CA, USA) using 1 nmol of each sense and antisense primers and 1 U of Platinum Taq DNA polymerase (Invitrogen Life Technologies, San Diego, CA, USA) in a final volume of 50 µL. To amplify the components of the chimeric proteins in transfected N1E-115 cells, we used Multiplex PCR along with 2 pairs of specific oligonucleotides for TCII and OLEO. For TCII, the forward primer was 5′-CATTGGGCATGATCACAAGGG-3′, and the reverse primer was 5′- GAGGAATGGTCTCAGCAGCTGG-3′ (GenBank access: NM_000355). For OLEO, the forward primer was 5′- TCACTTCTCGGAACCATAAT -3′, and the reverse primer was 5′- CCAGCATCCTTTGTCTTCTGCC-3′ (GenBank access: AY605694). In cells transfected with TCII the amplified fragment was of 551 bp, whereas the fragment was 275 bp in cell transfected with OLEO. In cells transfected with the chimeric proteins, the amplified fragments were 1347 bp for TCII-OLEO and 1240 bp for OLEO-TCII, which include the two components of the chimeric proteins. The internal control was a 349-bp-product of β-actin amplified using the forward primer 5′-CGTAAAGACCTCTATGCCAA-3′ and the reverse primer 5′-AGCCATGCCAAATGTCTCAT-3′. After an initial denaturation at 94°C for 2 min, amplification was done with 30 cycles as follows: denaturation, 94°C for 1 min; annealing, 59°C for multiplex PCR or 56°C for β-Actin; extension, 72°C for 1 min. Conventional PCR was used to amplify TCII-OLEO and OLEO-TCII products in the substantia nigra. To amplify a 380 bp fragment of TCII-OLEO, the forward primer was 5′-TTAGTCTCTTGCCGCCGTACAG-3′, and the reverse primer was 5′-ACCACCACTAACATCGTAGCCG-3′. To amplify a 394 bp fragment of OLEO-TCII the forward primer was 5′-TCACTTCTCGGAACCATAATCGG-3′ and the reverse primer was 5′-CCATCCAAGGTAAGAGGTGCTG-3′. After an initial denaturation at 94°C for 2 min, amplification was done with 30 cycles as follows: denaturation, 94°C for 1 min, annealing, 58°C for TCII-OLEO or 57°C for OLEO-TCII; extension, 72°C for 1 min. β-actin was used as internal control using the primers and PCR conditions described above. PCR products were analyzed by 2% agarose gel electrophoresis, stained with ethidium bromide, and photographed with a Kodak DC290 camera.

### Cell Viability

After transfection, N1E-115 cells seeded in 6-well dishes were selected with 800 µg/mL of G418 (Sigma-Aldrich, St. Louis, MO, US) for 15 days, and then transferred to 24-well dishes for viability studies. Cell viability was monitored using the colorimetric MTT (3-(4,5-dimethylthiazol-2-yl)-2,5-diphenyl tetrazolium bromide) assay to explore whether the protein expression could produce cytotoxicity in stably transfected N1E-115 cells. Briefly, after a 15-day selection period with G418, 6000 N1E-115 cells were seeded in 24-well plates and incubated with MTT (Roche Diagnostics Corporation; Indianapolis, IN, USA) at a final concentration of 0.5 mg/mL of incubation medium, for 4 h. Then, the solubilization solution (10% SDS, 0.01 M HCl) was added into the wells and incubated overnight. The total volume of each well was transferred to respective wells of an ELISA plate to determine the absorbance at 595/690 nm using a multiwell microtiter plate reader (Labsystems Multiskan, Multisoft, Helsinki, Finland).

### Western Blot

Proteins were extracted from the cell homogenates with Trizol following the manufacturer's protocol. Fifty micrograms of proteins quantified with Coomassie Plus assay kit (Pierce Biotechnology, Rockford, IL, USA) were subjected to electrophoresis in 10% SDS–PAGE gels and transferred onto nitrocellulose membranes (Amersham Bio-Sciences, Piscataway, NJ, USA). After blocking in 5% skim milk in 50 ml phosphate buffered saline solution (PBS; 8.1 mM Na_2_HPO_4_, 1.2 mM KH_2_PO_4_, 138 mM NaCl, 2.7 mM KCl, pH 7.4) – 0.1% Tween 20 for 1 h with shaking, membranes were incubated with either a goat polyclonal antibody to TCII (1∶8000 dilution; Santa Cruz Biotechnology, Santa Cruz, CA, USA), a rabbit cleaved Caspase-3 (1∶600; Cell Signaling Technology Inc., Danvers, MA), a mouse anti-p53 peroxidase-labeled antibody (1∶500 dilution; Santa Cruz Biotechnology) at 4°C overnight. Secondary antibodies (incubation 2 h at room temperature) were a peroxidase-labeled donkey anti-goat IgG (1∶20000 dilution; Zymed, San Francisco, CA, USA) and a peroxidase-labeled donkey anti-rabbit IgG (1∶10000 dilution; Amersham Bio-Sciences, Piscataway, NJ, USA). A Supersignal West pico chemiluminescence kit (Pierce Biotechnology, Rockford, IL, USA) and Kodak Biomax MR films were used for detection of the immunoreactivity. To normalize the total amount of protein per lane, membranes were stripped and incubated with a monoclonal mouse antibody against actin (1∶500 dilution; (CINVESTAV, Mexico)) and the peroxide-labeled goat anti-mouse IgG (1∶10000 dilution; Zymed, San Francisco, CA, USA). The immunoreactivity was detected by chemiluminescence.

### Immunofluorescence Assays

Using indirect immunofluorescence of TCII showed transgenic expression, whereas apoptosis was shown by indirect immunofluorescence of cleaved Caspase-3. Apoptosis and necrosis were explored in stably transfected N1E-115 cells at the 6th day in culture. The latter cells were incubated with 4 µM propidium iodide added in the culture medium to mark necrotic cells and 10 min later were fixed and immunostained against cleaved Caspase-3 to determine apoptosis. TCII and TH double immunofluorescence was used to show the transgenic expression in dopamine neurons. Cultured cells were washed once with PBS and fixed with 4% paraformaldehyde. Rats were deeply anesthetized with chloral hydrate and trans-cardially perfused through the ascending aorta with 100 ml of PBS, followed by 100 mL of 4% paraformaldehyde in PBS. The brains were dissected out and immersed in 4% paraformaldehyde at 4°C for 24 h. After an additional incubation in PBS containing 30% of sucrose at 4°C for 72 h, brains were frozen and sectioned into 40 µm slices on the coronal or sagital plane using the Leica CM1100 cryostat (Leica Microsystems; Nussloch, Germany). Permeabilization was made with 0.1% Triton X-100 in PBS for 30 min for cultured cells and 60 min for tissue sections, at room temperature (RT). After blocking the nonspecific binding sites with horse serum (5% for cultured cells and 10% for tissue sections) dissolved in PBS–0.1% Triton X-100 for 30 min at room temperature (RT), the cells were incubated with the primary antibody over night at 4°C. The primary antibodies were, a goat polyclonal antibody anti-TCII (1∶50 dilution; Santa Cruz Biotechnology, Santa Cruz, CA, USA), a mouse monoclonal antibody anti-human golgin-97 (1∶50 dilution; Molecular Probes Inc, Eugene, OR, USA), a rabbit polyclonal antibody anti-calreticulin (1∶100 dilution; Bioreagents, Golden, CO, USA), and a rabbit polyclonal cleaved anti-caspase-3 (1∶500 dilution; New England Biolabs, Beverly, MA, USA). After washing with PBS, cells and slides were incubated for 2 h at RT with the suitable secondary antibody. These antibodies were, a FITC-donkey anti-goat IgG (1∶60 dilution; Jackson ImmunoResearch; Palo Alto, CA, USA Palo Alto, CA, USA), a FITC- (1∶60 Sigma, St. Louis, MO, USA) or Rho-donkey anti-rabbit IgG (1∶60 Jackson ImmunoResearch; Palo Alto, CA, USA), and Rho- or AMCA-donkey anti-mouse IgG (1∶60 Jackson ImmunoResearch; Palo Alto, CA, USA), incubated for 2 h at RT. Finally, the cells and slices were washed and mounted on glass slides using Vectashield (Vector Laboratories, Burlingame, CA, USA). The fluorescence was observed with a Leica DMIRE2 microscope using the objectives 5X, 20X, and 40X, and the filters A for AMCA or Hoechst 33258, K3 for FITC, and TX2 for Rho. The images were digitized with a Leica DC300F camera (Nussloch, Germany). Negative controls were obtained by omitting the primary antibody, and replacing it by an irrelevant antibody of the same IgG subclass. Fluorescence labeling was also viewed through a multispectral confocal laser-scanning microscope (TCS-SPE, Leica, Heidelberg) using a 60X oil-immersion objective at excitation-emission wavelengths of 405–465 nm (blue channel), 488–522 nm (green channel), and 568–635 nm (red channel). Twenty to forty consecutive optical sections at 0.3-µm intervals were obtained in the z-series. The resulting images were projected on a bidimensional plane and were overlapped on the screen monitor using blue for Hoechst 33258, green for GFP and FITC, and red for TRITC.

### Behavioral Testing

Ipsilateral turning behavior caused by intraperitoneal injection of methamphetamine (8 mg/kg of body weight) was automatically assessed at 30 and 60 days after transfection by using a homemade device [23]. Rats were fitted around the chest with a Velcro harness connected to a flexible cable and placed in a cylindrical plastic container measuring 30 cm in diameter and 40 cm high. The cables were attached to a light bi-directional swivel switcher interfaced with a microcomputer. Number and direction of turns were evaluated automatically with the following algorithms: left direction, 0,1 interruptions; right direction, 1,0 interruptions; and turn counter, 1,1 interruptions. The rotational asymmetry score was expressed as full body turns per minute over a 90 min period. The open field test was performed as described [45].

### Statistical Analysis

All values are expressed as the mean±SEM. The repeated-measures two-way ANOVA was used to analyze the difference in cell viability and turning behaviors. The difference between pairs of means was shown by the Bonferroni post-test. The statistical analysis was done with the Graph Pad Software (San Diego, CA, USA). In all analyses, the null hypothesis was rejected at the 0.05 level.

## References

[1] Russell JSR, Batten FE, Collier J (1900). Subacute combined degeneration of the spinal cord.. Brain.

[2] Scalabrino G (2009). The multi-faceted basis of vitamin B12 (cobalamin) neurotrophism in adult central nervous system: Lessons learned from its deficiency.. Prog Neurobiol.

[3] Forges T, Monnier-Barbarino P, Alberto JM, Guéant-Rodriguez RM, Daval JL (2007). Impact of folate and homocysteine metabolism on human reproductive health.. Hum Reprod Update.

[4] Cheng H, Gomes-Trolin C, Aquilonius SM, Steinberg A, Löfberg C (1997). Levels of L-methionine S-adenosyltransferase activity in erythrocytes and concentrations of S-adenosylmethionine and S-adenosylhomocysteine in whole blood of patients with Parkinson's disease.. Exp Neurol.

[5] Fuso A, Seminara L, Cavallaro RA, D'Anselmi F, Scarpa S (2005). S-adenosylmethionine/homocysteine cycle alterations modify DNA methylation status with consequent deregulation of PS1 and BACE and beta-amyloid production.. Mol Cell Neurosci.

[6] Ho PI, Collins SC, Dhitavat S, Ortiz D, Ashline D (2001). Homocysteine potentiates beta-amyloid neurotoxicity: role of oxidative stress.. J Neurochem.

[7] Kruman II, Kumaravel TS, Lohani A, Pedersen WA, Cutler RG (2002). Folic acid deficiency and homocysteine impair DNA repair in hippocampal neurons and sensitize them to amyloid toxicity in experimental models of Alzheimer's disease, J. Neurosci.

[8] Blaise SA, Nedelec E, Schroeder H, Alberto JM, Bossenmeyer-Pourie C (2008). Gestational vitamin B deficiency leads to homocysteine-associated brain apoptosis and alters neurobehavioral development in rats.. Am J Pathol.

[9] Blaise S, Alberto JM, Nedelec E, Ayav A, Pourié G (2005). Mild neonatal hypoxia exacerbates long term effects of hyperhomocysteinemia in the rat pup.. Pediatric Research.

[10] Choi SW, Friso S, Ghandour H, Bagley PJ, Selhub J (2004). Vitamin B-12 deficiency induces anomalies of base substitution and methylation in the DNA of rat colonic epithelium.. J Nutr.

[11] Gharib A, Chabannes B, Sarda N, Pacheco H (1983). In vivo elevation of mouse brain S-adenosyl-L-homocysteine after treatment with L-homocysteine.. J Neurochem.

[12] Daval JL, Blaise S, Guéant JL (2009). Vitamin B deficiency causes neural cell loss and cognitive impairment in the developing rat.. Proc Natl Acad Sci U S A.

[13] Troen AM, Shea-Budgell M, Shukitt-Hale B, Smith DE, Selhub J (2008). B-vitamin deficiency causes hyperhomocysteinemia and vascular cognitive impairment in mice.. Proc Natl Acad Sci USA.

[14] Buccellato FR, Miloso M, Braga M, Nicolini G, Morabito A (1999). Myelinolytic lesions in spinal cord of cobalamin-deficient rats are TNF-alpha-mediated.. FASEB J.

[15] Brunaud L, Alberto JM, Ayav A, Gérard P, Namour F (2003). Vitamin B12 is a strong determinant of low methionine synthase activity and DNA hypomethylation in gastrectomized rats.. Digestion.

[16] Pons L, Battaglia-Hsu SF, Orozco-Barrios CE, Ortiou S, Chery C (2009). Anchoring secreted proteins in endoplasmic reticulum by plant oleosin: the example of vitamin B12 cellular sequestration by transcobalamin.. PLoS ONE. 2009 Jul 22;.

[17] Capuano F, Beaudoin F, Napier JA, Shewry PR (2007). Properties and exploitation of oleosins.. Biotechnol Adv.

[18] Rothenberg SP, Quadros EV (1995). Transcobalamin II and the membrane receptor for the transcobalamin II-cobalamin complex.. Baillieres Clin Haematol.

[19] Wuerges J, Garau G, Geremia S, Fedosov SN, Petersen TE (2006). Structural basis for mammalian vitamin B12 transport by transcobalamin.. Proc Natl Acad Sci U S A.

[20] McLean GR, Quadros EV, Rothenberg SP, Morgan AC, Schrader JW (1997). Antibodies to transcobalamin II block in vitro proliferation of leukemic cells.. Blood.

[21] Michalak M, Groenendyk J, Szabo E, Gold LI, Opas M (2009). Calreticulin, a multi-process calcium-buffering chaperone of the endoplasmic reticulum.. Biochem J.

[22] Tai G, Lu L, Johannes L, Hong W (2005). Functional analysis of Arl1 and golgin-97 in endosome-to-TGN transport using recombinant Shiga toxin B fragment.. Methods Enzymol.

[23] Hsu-Battaglia SF, Akchiche N, Noel N, Alberto JM, Jeannesson E (2009). Vitamin B12 deficiency reduces proliferation and promotes differentiation of neuroblastoma cells and upregulates PP2A, proNGF and TACE.. Proc Natl Acad Sci U S A, in press.

[24] Anaya-Martinez V, Martinez-Marcos A, Martinez-Fong D, Aceves J, Erlij D (2006). Substantia nigra compacta neurons that innervate the reticular thalamic nucleus in the rat also project to striatum or globus pallidus: implications for abnormal motor behavior.. Neuroscience.

[25] Scalabrino G, Carpo M, Bamonti F, Pizzinelli S, D'Avino C (2004). High tumor necrosis factor-alpha levels in cerebrospinal fluid of cobalamin-deficient patients.. Ann Neurol.

[26] De Lau LM, Koudstaal PJ, Witteman JC, Hofman A, Breteler MM (2006). Dietary folate, vitamin B12, and vitamin B6 and the risk of Parkinson disease Neurology.

[27] Todorovic Z (2006). Homocysteine serum levels and MTHFR C677T genotype in patients with Parkinson's disease, with and without levodopa therapy.. J Neurol Sci.

[28] Kuhn W, Roebroek R, Blom H, van Oppenraaij D, Przuntek H (1998). Elevated plasma levels of homocysteine in Parkinson's disease.. Eur Neurol.

[29] Muller T, Werne B, Fowler B, Kuhn W (1999). Nigral endothelial dysfunction, homocysteine, and Parkinson's disease.. Lancet.

[30] Mahfouz MM, Kummerow FA (2004). Vitamin C or vitamin B6 supplementation prevents the oxidative stress and decrease of prostacyclin generation in homocysteinemic rats.. Int J Biochem Cell Biol.

[31] Kannan K, Jain SK (2004). Effect of vitamin B6 on oxygen radicals, mitochondrial membrane potential, and lipid peroxidation in H2O2-treated U937 monocytes.. Free Radic Biol Med.

[32] Chumnantana R, Yokochi N, Yagi T (2005). Vitamin B6 compounds prevent the death of yeast cells due to menadione, a reactive oxygen generator.. Biochim Biophys Acta.

[33] Jain SK, Lim G (2001). Pyridoxine and pyridoxamine inhibits superoxide radicals and prevents lipid peroxidation, protein glycosylation, and (Na+ K+)-ATPase activity reduction in high glucose-treated human erythrocytes.. Free Radic Biol Med.

[34] Tan EK, Cheah SY, Fook-Chong S, Yew K, Chandran VR (2005). Functional COMT variant predicts response to high dose pyridoxine in Parkinson's disease.. Am J Med Genet B Neuropsychiatr Genet.

[35] Lamberti P, Zoccolella S, Armenise E, Lamberti SV, Fraddosio A (2005). Hyperhomocysteinemia in L-dopa treated Parkinson's disease patients: effect of cobalamin and folate administration.. Eur J Neur.

[36] Rogers JD, Sanchez-Saffon A, Frol AB, Diaz-Arrastia R (2003). Elevated plasma homocysteine levels in patients treated with levodopa.. Arch Neurol.

[37] Yasui K, Nakaso K, Kowa H, Takeshima T, Nakashima K (2003). Levodopa-induced hyperhomocysteinaemia in Parkinson's disease.. Acta Neurol Scand.

[38] Miller JW, Shukitt-Hale B, Villalobos-Molina R, Nadeau MR, Selhub J (1997). Effect of L-dopa and the catechol-O-methyltransferase inhibitor Ro 41-0960 on sulphur amino acid metabolites in rats.. Clin Neuropharmacol.

[39] Smith AD, Refsum H (2009). Vitamin B-12 and cognition in the elderly.. Am J Clin Nutr.

[40] Spada RS, Stella G, Calabrese S, Bosco P, Anello G (2007). Association of vitamin B12, folate and homocysteine with functional and pathological characteristics of the elderly in a mountainous village in Sicily.. Clin Chem Lab Med.

[41] Rubio-Zapata HA, Rembao-Bojorquez JD, Arango-Rodriguez ML, Dupouy S, Forgez P (2009). NT-polyplex: a new tool for therapeutic gene delivery to neuroblastoma tumors.. Cancer Gene Ther.

[42] Gonzalez-Barrios JA, Lindahl M, Bannon MJ, Anaya-Martinez V, Flores G (2006). Neurotensin polyplex as an efficient carrier for delivering the human GDNF gene into nigral dopamine neurons of hemiparkinsonian rats.. Mol Ther.

[43] Martinez-Fong D, Navarro-Quiroga I (2000). Synthesis of a non-viral vector for gene transfer via the high-affinity neurotensin receptor.. Brain Res Brain Res Protoc.

[44] Navarro-Quiroga I, Gonzalez-Barrios JA, Barron-Moreno F, Gonzalez-Bernal V, Martinez-Arguelles DB (2002). Improved neurotensin-vector-mediated gene transfer by the coupling of hemagglutinin HA2 fusogenic peptide and Vp1 SV40 nuclear localization signal.. Brain Res Mol Brain Res.

[45] Arango-Rodriguez ML, Navarro-Quiroga I, Gonzalez-Barrios JA, Martinez-Arguelles DB, Bannon MJ (2006). Biophysical characteristics of neurotensin polyplex for in vitro and in vivo gene transfection.. Biochim Biophys Acta.

[46] Raffo E, de Vasconcelos AP, Boehrer A, Desor D, Nehlig A (2009). Neurobehavioral maturation of offspring from epileptic dams: Study in the rat lithium-pilocarpine model.. Exp Neurol.

